# The Clinical Outcomes of Locally Advanced Cervical Esophageal Squamous Cell Carcinoma Patients Receiving Curative Concurrent Chemoradiotherapy: A Population-Based Propensity Score-Matched Analysis

**DOI:** 10.3390/cancers11040451

**Published:** 2019-03-30

**Authors:** Yen-Hao Chen, Hung-I Lu, Chien-Ming Lo, Yu-Ming Wang, Shang-Yu Chou, Chang-Chun Hsiao, Shau-Hsuan Li

**Affiliations:** 1 Department of Hematology-Oncology, Kaohsiung Chang Gung Memorial Hospital and Chang Gung University College of Medicine, Kaohsiung 833, Taiwan; alex8701125@gmail.com; 2 Graduate Institute of Clinical Medical Sciences, College of Medicine, Chang Gung University, Taoyuan 333, Taiwan; cchsiao@mail.cgu.edu.tw; 3 School of Medicine, Chung Shan Medical University, Taichung 402, Taiwan; 4 Department of Thoracic & Cardiovascular Surgery, Kaohsiung Chang Gung Memorial Hospital and Chang Gung University College of Medicine, Kaohsiung 833, Taiwan; luhungi@yahoo.com.tw (H.-I.L.); t123207424@cgmh.org.tw (C.-M.L.); 5 Department of Radiation Oncology, Kaohsiung Chang Gung Memorial Hospital and Chang Gung University College of Medicine, Kaohsiung 833, Taiwan; scorpion6088@gmail.com (Y.-M.W.); a9682@cgmh.org.tw (S.-Y.C.); 6 Division of Pulmonary and Critical Care Medicine, Kaohsiung Chang Gung Memorial Hospital, Kaohsiung 833, Taiwan

**Keywords:** esophageal cancer, squamous cell carcinoma, concurrent chemoradiotherapy, propensity score matching

## Abstract

This study investigated the clinical outcome of locally advanced cervical esophageal squamous cell carcinoma (ESCC) patients who received curative concurrent chemoradiotherapy (CCRT) and their differences from thoracic ESCC patients. Among 411 enrolled ESCC patients, including 63 with cervical and 348 with thoracic ESCC, 63 thoracic patients were propensity score-matched to the 63 cervical patients. For cervical ESCC, T4b and high tumor grade were independent prognostic factors of a worse overall survival (OS) in univariate and multivariate analyses. The response rates to curative CCRT between cervical and the matched thoracic ESCC groups were similar but cervical ESCC had a better OS than that of the matched thoracic group (21.4 versus 10.1 months, *p* = 0.012). Better OS was mentioned to be in the patients with complete response (CR), whether in the cervical or matched thoracic ESCC group. For patients without CR, patients who underwent esophagectomy had superior OS than those without operation in the matched thoracic ESCC group (11.6 versus 11.9 months, *p* = 0.73). Only three patients received operation in the cervical ESCC group, thus the survival difference was not significant. Curative CCRT may be a reasonable treatment for cervical ESCC in clinical practice, and the role of surgery should be considered as salvage therapy if residual disease is evident.

## 1. Introduction

Esophageal cancer is male-predominant and one of the most aggressive malignancies worldwide. In Taiwan, more than 90% of esophageal cancer patients are diagnosed with esophageal squamous cell carcinoma (ESCC), which ranks ninth as a cause of cancer-related mortality [1]. According to anatomic tumor location, esophageal cancer is divided into cervical or thoracic esophageal cancer. The cervical esophagus is only 5 cm in length and cervical ESCC is a small population of patients, accounting for less than 10% of all esophageal cancer patients [2]. For operable esophageal cancer, neoadjuvant chemotherapy or chemoradiotheapy followed by esophagectomy is the gold-standard therapeutic modality. For cervical ESCC, total laryngoesophagetomy with gastric pull-up reconstruction or colon interposition is frequently indicated; however, this surgical procedure is relatively complicated, resulting in a high postoperative mortality rate (around 10%), poor five-year overall survival (OS) rate (around 20%), increased significant morbidities, and decreased quality of life [3].

In contrast, concurrent chemoradiotherapy (CCRT) is the standard treatment for inoperable esophageal cancer and growing evidence suggests that CCRT improves the response and OS rates of these esophageal cancer patients [4,5,6,7,8]. A Japanese study showed that CCRT had sufficient overall response rate and safety in patients with cervical ESCC [8]. Bedenne et al. reported a higher OS and lower three-month mortality rates and cumulative hospital stay in the chemoradiotherapy (CRT) group compared to those of the CRT followed by surgery group among esophageal cancer patients [5]. Another study in Germany demonstrated no significant differences in outcomes and patterns of failure between neoadjuvant CRT plus surgery and definitive CRT [9]. The Cochrane Database of Systemic Reviews shows that the addition of surgery to CRT makes no difference in OS and may be related to higher treatment-related mortality compared to that of CRT alone in locally advanced ESCC patients [10]. Therefore, in comparison to radical esophagectomy, definitive CCRT is favored by some physicians for locally advanced inoperable esophageal cancer in clinical practice [6]. However, to the best of our knowledge, limited studies have focused on the outcomes of cervical ESCC patients who receive CCRT with curative intent.

The current study retrospectively reviewed data from locally advanced cervical ESCC patients who received CCRT with curative intent in our hospital. The aim of the present study was to investigate the clinical outcomes of locally advanced cervical ESCC patients who received curative CCRT and their differences from those of thoracic ESCC patients.

## 2. Results

### 2.1. Patient Characteristics

A total of 411 patients with locally advanced ESCC who received curative CCRT at Kaohsiung Chang Gung Memorial Hospital who matched the eligibility criteria were included in this study, including 348 and 63 patients with thoracic and 63 cervical ESCC, respectively. To prevent selection bias, we identified 63 matched patients among the 348 thoracic ESCC patients using the propensity score matching method to compare to the 63 cervical ESCC patients. The parameters, including age, gender, T status, N status, tumor stage, and tumor grade, were all matched and there were no statistical differences between these two groups. The baseline characteristics of the patients with cervical and thoracic ESCC are summarized in Table 1.

### 2.2. Clinical Outcomes of Cervical ESCC Patients

Among the 63 cervical ESCC patients, 61 were male and 2 were female, with a median age of 58 years (range, 37–80 years). With respect to OS, there were no significant differences in terms of age (<60 versus ≥60 years), N status (N0–1 versus N2–3), and tumor stage (stage IIIA–B versus stage IIIC) in a univariate analysis. Meanwhile, the 19 patients with non-T4b (T2-4a) status had a significantly better OS than that of the 44 patients with T4b disease (26.1 versus 17.3 months, *p* = 0.035). The 50 patients with grade 1–2 disease had a superior OS compared to that of the 13 patients with grade 3 disease (22.2 versus 11.3 months, *p* = 0.015). Multivariate analysis showed that non-T4b status (*p* = 0.044, hazard ratio: 0.47, 95% confidence interval: 0.23–0.98) and grade 1–2 disease (*p* = 0.023, hazard ratio: 0.42, 95% confidence interval: 0.20–0.89) remained the independent prognostic factors of a superior OS. The results of univariate and multivariate analyses of OS in 63 cervical ESCC patients are summarized in Table 2. 

### 2.3. Comparisons between Cervical and Thoracic ESCC Patients

The 411 stage III locally advanced ESCC patients who received CCRT with curative intent were divided into the cervical (N = 63) and thoracic (N = 348) ESCC groups. There were no significant differences between these two groups, except for T status (*p* = 0.001), N status (*p* = 0.023), and tumor stage (*p* = 0.012). The cervical ESCC group had significantly more patients with advanced T status and tumor stage in compared to those the thoracic ESCC group. To prevent selection bias, 63 matched ESCC patients among the 348 thoracic patients were selected using a propensity score matching method; these parameters were all matched without statistical difference between these two groups (Table 1). The median dose of radiotherapy was 66 Gy (range: 66–70) for cervical ESCC and 50.4 Gy (range: 50–50.4) for thoracic ESCC. There were 61 and 60 patients who received chemotherapy with cisplatin/5-fluorouracil in the cervical and matched thoracic ESCC groups, respectively; carboplatin/5-fluorouracil was prescribed for two patients in the cervical ESCC group and three patients in the matched thoracic group. Compared to the matched thoracic ESCC group, there was a higher complete response (CR) rate of CCRT in the cervical ESCC group (33% versus 16%, *p* = 0.038) but the response (CR + partial response (PR)) and disease control (CR + PR + stable disease (SD)) rates were similar, without significant differences between these two groups (Table 3). 

**Figure 1:**
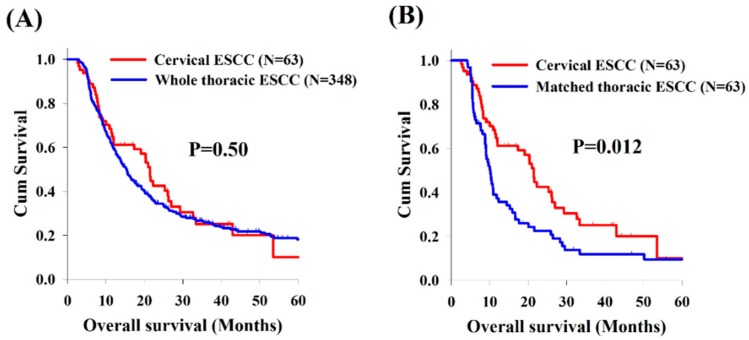
Kaplan–Meier curves comparing overall survival between patients with cervical and thoracic esophageal squamous cell carcinoma (ESCC). (**A**) Cervical ESCC group versus whole thoracic ESCC group. (**B**) Cervical ESCC group versus matched thoracic ESCC group.

**Figure 2:**
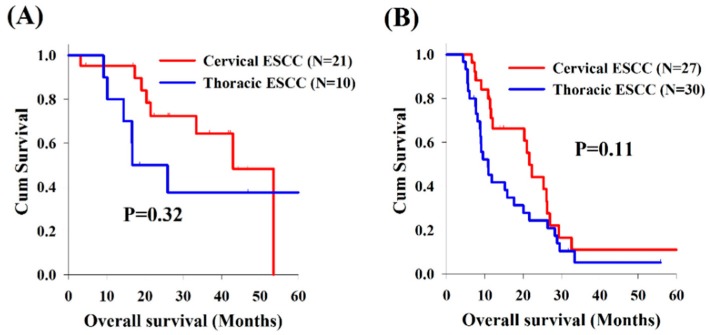

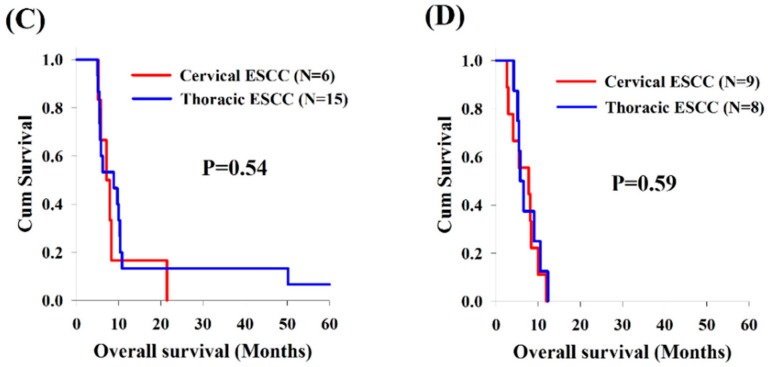
Comparison of overall survival between patients with cervical and thoracic esophageal squamous cell carcinoma according to treatment responses: (**A**) complete response; (**B**) partial response; (**C**) stable disease; and (**D**) progressive disease.

Analysis of the OS revealed no significant differences between cervical and whole thoracic ESCC groups (Figure 1A). However, compared to matched thoracic ESCC group, the cervical ESCC group had a significantly superior OS (21.4 versus 10.1 months, *p* = 0.012, Figure 1B). In addition, there were no significant differences in OS between these two groups according to treatment response (CR, PR, SD, and progressive disease [PD]) (Figure 2).

### 2.4. The Effect of Surgical Intervention

**Figure 3:**
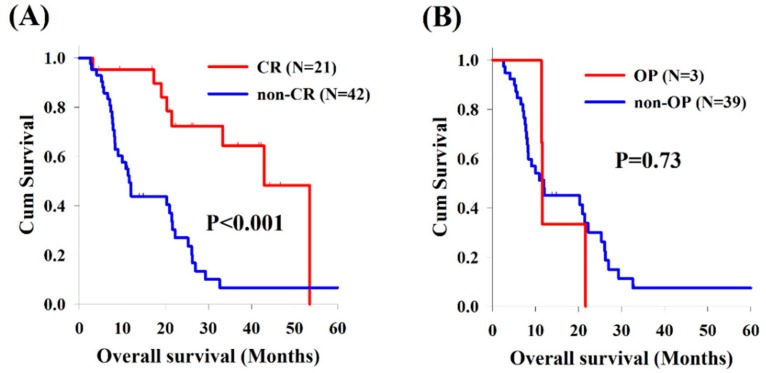
Kaplan–Meier survival curves of overall survival among patients with cervical esophageal squamous cell carcinoma: (**A**) CR group versus non-CR group; and (**B**) OP versus non-OP in the non-CR group. CR, complete response; OP, operation.

In the analysis of cervical ESCC, the cervical ESCC group included 21 patients (33%) with CR and 42 patients (67%) without CR after CCRT, and the median OS was superior to that of the 21 patients who got CR (42.9 versus 11.6 months, *p* < 0.001, Figure 3A). Moreover, there were only three patient who received esophagectomy among these 42 patients without CR; however, there was no significant difference of OS between these patients with or without operation (11.6 versus 11.9 months, *p* = 0.73, Figure 3B).

**Figure 4:**
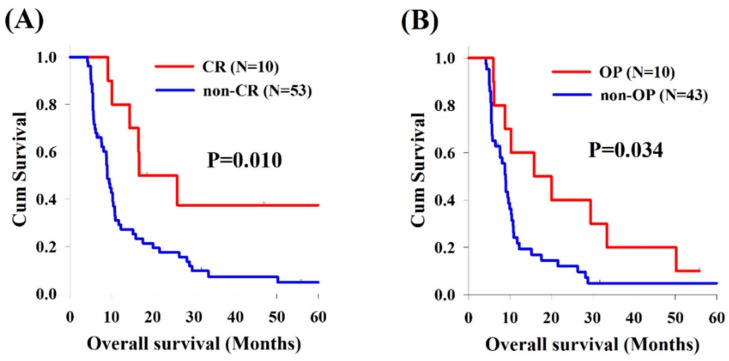
Kaplan–Meier survival curves of overall survival among patients with thoracic esophageal squamous cell carcinoma: (**A**) CR group versus non-CR group; and (**B**) OP versus non-OP in the non-CR group. CR, complete response; OP, operation.

With respect to thoracic ESCC, there were 10 patients (16%) with CR and 53 patients (84%) without CR in the matched thoracic ESCC group, and better OS was found to be in the CR group compared to the non-CR group (16.6 versus 9.0 months, *p* = 0.010, Figure 4A). In the non-CR group, 10 patients who underwent salvage esophagectomy had superior OS than those without operation (15.9 versus 9.0 months, *p* = 0.034, Figure 4B).

## 3. Discussion

Cervical esophageal cancer accounts for only 5–10% of all esophageal cancer cases, while squamous cell carcinoma is the major histology [2,11]. Cervical ESCC is often locally advanced at initial presentation, with infiltration to nearby structures such as the hypopharynx, larynx, trachea, and great vessels, resulting in increased treatment difficulty and risk of complications [2,11]. In the past, radical esophagectomy with reconstruction was the gold-standard therapeutic modality for operable cervical ESCC patients. However, this complicated surgical procedure contributed to high mortality and morbidity, resulting in a poor OS and quality of life [3]. Recently, growing evidence has shown that the survival benefit of definitive CRT is equal to that of radical surgery in cervical ESCC patients. A Japanese study reported by Takebayashi et al. demonstrated that CRT and curative surgery as initial treatment have comparable outcomes in cervical esophageal cancer patients [12]. Another cohort study from Italy compared the outcomes of three common treatment strategies, namely surgery alone, CRT followed by surgery, and definitive CRT, concluding that definitive CRT should be the treatment of choice for cervical esophageal cancer and that surgery supports the survival benefit among patients with non-complete response [13]. Therefore, definitive CCRT rather than radical surgery for cervical ESCC patients is more and more indicated in clinical practice [6,14]. To the best of our knowledge, few studies have focused on the prognosis of cervical ESCC; therefore, the present study was designed to evaluate the prognostic factors in cervical ESCC patients in comparison to those in thoracic ESCC patients.

In the current study, T4b status and high tumor grade of differentiation were both independent poor prognostic factors in univariate and multivariate analyses. For ESCC patients, a T4b status is considered inoperable as initial presentation and definitive CRT is more feasible for these patients. Previous studies have shown that surgical resection as an initial treatment strategy does not improve the outcome of esophageal cancer patients with T4b status [15,16,17]. Ishikawa et al. demonstrated that definitive CRT is more favorable for unresectable T4b ESCC and contributed to downstaging from unresectable to resectable disease in 70% of ESCC patients [18]. Another Japanese study that enrolled 11 patients with advanced cervical ESCC, including those with T4b status, revealed that definitive CRT with docetaxel/cisplatin/5-fluorouracil contributed to a CR in 10 patients (91%) but 50% of patients with a CR later experienced a tumor recurrence [19]. In our study, 11 patients with T4b achieved a CR after CCRT among 44 cervical ESCC patients with T4b status, corresponding to a CR rate of 25% and a median OS of 20.3 months. Although definitive CRT may lead to downstaging, the possibility of surgical resection, and CR, T4b status remains a poor prognostic factor. 

The goal of management for cervical ESCC is different from that of thoracic ESCC. Due to high mortality and morbidity caused by surgical intervention, curative CCRT is more a feasible and preferred treatment plan for cervical ESCC patients in clinical practice. To improve the cure rate, a higher dose of radiotherapy with 66–70 Gy is planned to increase the CR rate of these patients. In contrast, CCRT followed by esophagectomy is the gold-standard treatment for thoracic ESCC, with an average of radiotherapy dose of around 50 Gy. In the current study, the CR rate was higher in the cervical ESCC group compared to that of the matched thoracic ESCC group, although the response rates were similar between these two groups. In contrast, the cervical ESCC group had a superior OS compared to the matched thoracic ESCC group; these results may be associated with the higher dose of radiotherapy and higher number of patients achieving a CR.

Our study showed that better OS was mentioned to be in the patients with CR, whether in the cervical or thoracic ESCC group. For patients without CR, patients who underwent surgical resection had a better OS than those without operation in the thoracic group; however, for cervical ESCC group, there was no significant difference of OS between patients who underwent surgery and those who did not, although there may be bias because only 7% of cervical ESCC patients without CR underwent esophagectomy after CCRT. In contrast, 19% of patients without CR was found to receive salvage esophagectomy in the thoracic group. Although the benefit of surgical resection differed between groups, this may be related to very small number of patients who underwent operation in the cervical group; however, the role of surgery is still very important and should be considered for ESCC patients if residual disease is evident.

The major limitations of our current study include its retrospective design, the single institute, and the small number of patients enrolled despite the propensity score matching to prevent selection bias. A large randomized controlled trial involving a sufficient number of patients is warranted to confirm our observations and clarify the situation for cervical ESCC.

## 4. Material and Methods

### 4.1. Patient Eligibility and Study Design

The current study was approved by the Chang Gung Medical Foundation Institutional Review Board (201800845B0) and written informed consent was not necessary due to the retrospective design. From January 2005 to December 2015, patients initially diagnosed with ESCC and who underwent treatment at Kaohsiung Chang Gung Memorial Hospital were retrospectively reviewed. All enrolled patients were required to meet the following eligibility criteria: (1) Eastern Cooperative Oncology Group performance status 0–1; (2) confirmed esophageal cancer by pathological diagnosis, with only squamous cell carcinoma indicated, and excluding other pathological types; (3) stage III locally advanced disease without neck/celiac lymph node metastasis or distant metastasis; (4) only patients with isolated cervical or thoracic ESCC, and excluding those with synchronous or metachronous cervical and thoracic ESCC; and (5) no history of second primary malignancy such as head and neck cancers. Finally, a total of 411 ESCC patients were identified for further analysis, including 63 and 348 cervical and thoracic ESCC patients, respectively.

In our study, positron emission tomography (PET) scans, chest computed tomography (CT) and endoscopic ultrasonography (EUS) were essential pre-treatment examinations arranged for each patient, and the clinical tumor stage was determined according to the 7th American Joint Committee on Cancer (AJCC) staging system [20]. Cervical esophageal cancer was defined as a tumor located in the neck, approximately 15–20 cm in length from the incisors, with superior border by the hypopharynx and inferior border by the thoracic inlet (sternal notch) [21].

**Figure 5:**
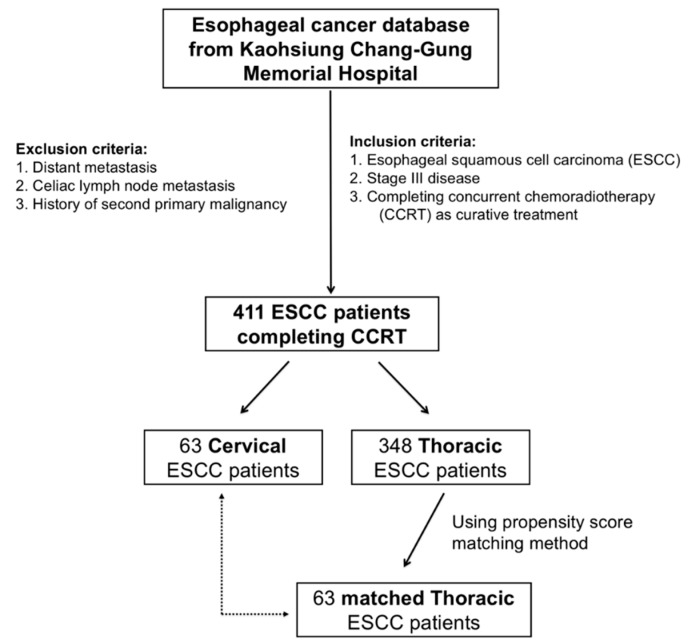
Algorithm for selecting patients with cervical and thoracic esophageal squamous cell carcinoma (ESCC).

Propensity score matching was used to avoid selection bias between the cervical and thoracic ESCC groups. First, a binary logistic regression with covariates including tumor T status, tumor N status, tumor stage, tumor grade, and patient age and gender were entered into the propensity model to calculate a propensity score. After that, a one-to-one match with the closest matching scores between the 63 cervical ESCC patients and 63 thoracic ESCC patients was established. The algorithm is shown in Figure 5.

### 4.2. CCRT Setting and Surgery

Local radiotherapy was prescribed with curative intent using intensity-modulated radiotherapy (IMRT) for each patient. For planning image acquisition, the patients were placed in a supine position and immobilized under customized thermoplastic casts. Then, CT simulations were used to acquire images with slice thicknesses of 3–5 mm. The treating radiation oncologist then delineated the targets on the CT images. The gross tumor and lymph nodes (LNs) shown on the CT scan and/or PET-CT were defined as the gross tumor volume (GTV); the esophagus, the mediastinum, and bilateral supraclavicular fossa were comprehensively defined as the clinical target volume (CTV). For cervical esophageal cancer patients, the bilateral neck was also included in the CTV. The planning target volumes (PTVs) for inverse IMRT planning were generated from the corresponding CTVs with 0.5–1.0 cm volumetric expansion. The prescribed dose to the PTV was 66–70 Gy in 33–35 daily fractions for cervical ESCC and 50–50.4 Gy in 25–28 daily fractions for thoracic ESCC. For some thoracic ESCC patients who initially presented with gross neck LNs, an additional dose of 10–16 Gy in 5–8 daily fractions was prescribed as a local boost.

Chemotherapy was administered concurrently with radiotherapy and consisted of cisplatin at a dose of 75 mg/m^2^ via a 4-h intravenous drip infusion on Day 1, and 5-fluorouracil at 1000 mg/m^2^ via continuous intravenous drip infusion on Days 1–4 every 4 weeks. Carboplatin was administered instead of cisplatin in patients with creatinine clearance rates <60 mL/min. Patients underwent at least two cycles of chemotherapy during radiotherapy. The above-mentioned technique was performed as previously described [22,23,24,25,26].

The definition of “complete CCRT” is that patients must complete the planned radiotherapy without interruption more than three days and receive at least 2 cycles of chemotherapy.

Surgery was considered and performed for patients with ESCC who completed curative CCRT and were feasible for operation, and the role of surgery was defined as salvage procedure for cervical and thoracic ESCC patients with residual and resectable tumor.

### 4.3. Definition of Clinical Complete Response

The responses to CCRT treatment were assessed by EUS, chest CT, and PET for each patient and defined as a CR, PR, SD, or PD. The definitions of responses were determined according to the Response Evaluation Criteria in Solid Tumors (RECIST) guidelines and previous studies [27,28,29,30]. In terms of clinical CR, with respect to the primary esophageal tumor, no obvious endoscopic finding suggesting the presence of a tumor was mentioned by endoscopy and there was no evidence of residual malignancy by endoscopic biopsy. With respect to lymph nodes, reduction in the short axis of lymph nodes to <10 mm on chest CT and EUS was documented. In addition, there was no increased uptake of ^18^F-fluorodeoxyglucose in PET scans with maximum standardized uptake values of >3.4 and >4.1 for primary esophageal tumor and lymph nodes, respectively [31,32].

### 4.4. Statistical Analysis

The statistical analyses were performed using IBM SPSS Statistics, version 19.0 (IBM Corp., Armonk, NY, USA). Chi-square tests were used to assess the differences between groups for categorical variables, while multivariate analyses to determine the independent prognostic factors were computed with a Cox proportional hazards model. The definition of OS was the duration from the date of diagnosis to the death from any cause or most recent follow-up. Kaplan–Meier curves and log-rank tests were used to estimate the OS and the differences between the two groups, respectively. Statistical significance was defined as a two-sided *p*-value of 0.05.

## 5. Conclusions

The results of our study indicate that T4b status and high tumor grade are independent prognostic factors of locally advanced cervical ESCC patients and that CCRT is a reasonable curative treatment for cervical ESCC patients in clinical practice. The role of surgery should be considered as salvage therapy if residual disease is evident.

## Figures and Tables

**Table 1:** The characteristics in 411 patients with locally advanced stage III cervical and thoracic esophageal squamous cell carcinoma who received curative concurrent chemoradiotherapy.

Characteristics	Cervical ESCC Group (N = 63)	Thoracic ESCC Group (N = 348)	*p* Value
Age (years)			
<60 years	37 (59%)	242 (70%)	0.09
≥60 years	26 (41%)	106 (30%)	
Sex			
Male	61 (97%)	340 (98%)	0.68
Female	2 (3%)	8 (2%)	
T status			
1	0 (0%)	13 (4%)	0.001 *
2	1 (1%)	13 (4%)	
3	13 (21%)	144 (41%)	
4a	5 (8%)	38 (11%)	
4b	44 (70%)	140 (40%)	
N status			
0	5 (8%)	5 (2%)	0.023 *
1	24 (38%)	137 (39%)	
2	23 (37%)	136 (39%)	
3	11 (17%)	70 (20%)	
Tumor stage			
IIIA	7 (11%)	80 (23%)	0.012 *
IIIB	5 (8%)	54 (16%)	
IIIC	51 (81%)	214 (61%)	
Grade			
1	6 (12%)	61 (18%)	0.63
2	18 (35%)	203 (58%)	
3	27 (53%)	84 (24%)	
**Characteristics**	**Cervical ESCC Group (N = 63)**	**Thoracic ESCC Group # (N = 63)**	***p*-Value**
Age (years)			
<60 years	37 (59%)	37 (59%)	1.0
≥60 years	26 (41%)	26 (41%)	
Sex			
Male	61 (97%)	61 (97%)	1.0
Female	2 (3%)	2 (3%)	
T status			
1	0 (0%)	0 (0%)	1.0
2	1 (1%)	1 (1%)	
3	13 (21%)	13 (21%)	
4a	5 (8%)	5 (8%)	
4b	44 (70%)	44 (70%)	
N status			
0	5 (8%)	5 (8%)	1.0
1	24 (38%)	24 (38%)	
2	23 (37%)	23 (37%)	
3	11 (17%)	11 (17%)	
Tumor stage			
IIIA	7 (11%)	7 (11%)	1.0
IIIB	5 (8%)	5 (8%)	
IIIC	51 (81%)	51 (81%)	
Grade			
1	6 (12%)	6 (12%)	1.0
2	18 (35%)	18 (35%)	
3	27 (53%)	27 (53%)	

ESCC, esophageal squamous cell carcinoma; # using propensity score matching method; * statistically significant.

**Table 2:** Univariate and multivariate analysis of overall survival in 63 patients with locally advanced stage III cervical esophageal squamous cell carcinoma who received curative concurrent chemoradiotherapy.

Characteristics	No. of Patients	Univariate Analysis		Multivariate Analysis	
		Median OS (Months)	*p*-Value	HR (95% CI)	*p*-Value
Age (years)					
<60 years	37 (59%)	21.6	0.44		
≥60 years	26 (41%)	12.0			
T status					
2 + 3 + 4a	19 (30%)	26.1	0.035 *	0.47 (0.23–0.98)	0.044 *
4b	44 (70%)	17.3			
N status					
0 + 1	29 (46%)	21.6	0.54		
2 + 3	34 (54%)	19.1			
Tumor stage					
IIIA + IIIB	12 (19%)	25.3	0.56		
IIIC	51 (81%)	21.0			
Grade					
1 + 2	50 (79%)	22.2	0.015 *	0.42 (0.20–0.89)	0.023 *
3	13 (21%)	11.3			

OS, overall survival; HR, hazard ratio; CI, confidence interval; * statistically significant.

**Table 3:** Treatment response in 126 patients with locally advanced stage III cervical and thoracic esophageal squamous cell carcinoma who received curative concurrent chemoradiotherapy.

Response	Cervical ESCC Group (N = 63)	Thoracic ESCC Group # (N = 63)	*p*-Value
Complete response (CR)	21 (33%)	10 (16%)	
Partial response (PR)	27 (43%)	30 (48%)	
Stable disease (SD)	6 (10%)	15 (23%)	
Progressive disease (PD)	9 (14%)	8 (13%)	
CR rate	21 (33%)	10 (16%)	0.038 *
Response rate			
CR + PR	76%	64%	0.12
Disease control rate			
CR + PR + SD	83%	87%	0.62

ESCC, esophageal squamous cell carcinoma; # using propensity score matching method; * statistically significant.
